# The cost of protecting resources: a cross-sectional study on the interaction between LMX and role ambiguity on work addiction and burnout among Canadian first-level healthcare managers

**DOI:** 10.3389/fpsyg.2024.1298001

**Published:** 2024-04-23

**Authors:** Francis Maisonneuve, Patrick Groulx, Anaïs Galy, Denis Chênevert, Michel Cossette

**Affiliations:** HEC Montréal, Université de Montréal, Montreal, QC, Canada

**Keywords:** role ambiguity, work addiction, LMX, burnout, healthcare, managers

## Abstract

**Introduction:**

Using the conservation of resources (COR) theory, our study explores the interaction between role ambiguity and leader-member exchange (LMX) quality on burnout using work addiction as a mediator among Canadian first-level healthcare managers.

**Methods:**

Cross-sectional data was collected among 165 first-level managers working in healthcare with the support of interprofessional associations in Canada. Linear regression was used to test the presented hypotheses.

**Results:**

Work addiction fully mediated the positive relationship between role ambiguity and burnout among first-level managers. In addition, high LMX exacerbated both the direct and indirect effects of role ambiguity.

**Conclusion:**

Our study contributes by identifying role ambiguity as a context under which LMX can have adverse effects for first-level managers in healthcare. Moreover, work addiction acted as a mediator, theorized as a risky resource investment which depletes managers’ resources. Having a good relationship with their team further entices managers to develop a pathological relationship with their work to protect its members, which in turn is related to higher levels of burnout.

## Introduction

Role ambiguity poses crucial challenges for managers, such as reducing their ability to manage properly (Evans, 2016) or increasing their risk of experiencing burnout (Wu et al., 2019). More specifically, first-level managers (individuals with a supervisory role without managing other managers) are a population who could be particularly affected by role ambiguity as they are often thrusted into a management role without any real training or support to prepare them for it. Learning what are the expectations from their new managers can be an arduous task which is added to their maintained operational roles (Delaye and Boudrandi, 2010). When facing role ambiguity, first-level manager may be tempted to overinvest themselves into work to compensate the absence of clear expectations (Andreassen et al., 2019). Ambiguity can entice managers to work more while also expanding excessive effort during worktime to cover more potential goals. However, this approach puts them at risk of developing a pathological relationship with their work, namely work addiction (Andreassen, 2014), defined as “the compulsive and uncontrollable need to work incessantly” (Oates, 1971, p. 1).

The proposed phenomenon might be especially important to study in healthcare due to the specificities of this neuralgic sector (Johns, 2006). For instance, first-level healthcare managers are required to manage increasingly diverse teams while dealing with decreasing resources and constantly evolving expectations regarding their leadership (Gifford et al., 2023), contributing to ambiguity. Furthermore, these challenges emerge in an emotionally demanding setting as managers and team members must interact with individuals in ill health, increasing the risk of burnout (Maslach and Jackson, 1981). Yet, healthcare managers remain an understudied population regarding the emergence of burnout (Khammissa et al., 2022).

Studying burnout in healthcare is not new as it harkens back to the creation of its original measurement (Maslach and Jackson, 1981). However, developing new knowledge regarding the phenomenon remains important as healthcare systems across the world face multiple challenges. For instance, labor shortages fueled by burnout and high turnover are recurring issues in this sector (Willard-Grace et al., 2019). Furthermore, managing a team is already a heavily demanding position and we propose that acting as a leader in a healthcare context represents a unique challenge. Doubly so specifically for first-level managers who are also acting as caregivers. Providing quality care requires time, communication (DeVoe et al., 2002), and feeling well enough to do so (Ferrell, 2008). Those roles are in addition to managing their team and acting as a contact for middle-management with little to no training as a leader. Additionally, oftentimes in healthcare settings, first-level managers are former caregivers that did not necessarily received a proper managerial training when they accepted their new organizational role. Consequently, this can lead to a perception of blurred work expectations from the demands of intermediate and top-level management teams. Unclear expectations and objectives from team members or organizational leaders can generate stress for managers (Yoshie et al., 2008), especially if the demands from those groups are at odds. Further, these issues are particularly salient in healthcare settings due to potential misalignment between governmental inquiries focussing on organizational performance and caregivers demands originating from a will to provide both safe and high-quality care (Gross et al., 2007). Finally, the Canadian health care system, in which the study was conducted, is a public system funded by taxpayer and provides universal access to health care and social services. In this health care system, first-level managers are often expected to manage pluri-disciplinary teams, adding further complexity and potential ambiguity in their day-to-day tasks. As managers who themselves do not manage other managers, they maintain some operational obligations while also having to endorse a strategic role, artificially increasing the potential ambiguity of the objectives and expectations bestowed upon them. Accordingly, the proposed theoretical model with respect to role ambiguity appears to be highly relevant to be studied in a healthcare context.

A growing body of research is exploring potential underlying mechanisms relating ambiguity and burnout (e.g., Rubino et al., 2009; Dasgupta, 2012). However, to our knowledge, few studies theorized how role ambiguity affects resource investment strategies at work which in turn affects burnout. As such, we propose a theoretical model using the conservation of resources (COR) theory (Hobfoll, 1989, 2001) regarding work addiction. Under a resource perspective, we suggest that to compensate for uncertain objectives, first-level managers need to excessively invest resources (Clark et al., 2020), which can potentially lead to a pathological relationship with their work: work addiction (Andreassen, 2014). Compulsive working in turn exhausts resources, increasing the risk of burnout (Maslach et al., 1997). In summary, ambiguity-induced overexertion at work acts as a resource threat for first-level managers, a risk factor regarding burnout (Hobfoll et al., 2018).

Additionally, we expect this to be especially true when first-level managers have good leader-member exchange (LMX) with their subordinates, aiming to protect their team from ambiguity by overinvesting themselves further. As established by the COR theory (Hobfoll, 1989), individuals must invest resources to develop and protect their resources. When managers perceive to have quality relationships with their team members, high LMX, they perceive such relationships as a resource worth protecting (Uhl-Bien et al., 2022). In this context, LMX is not conceptualized as a resource threat, but as a catalyst which can exacerbate the relationship between ambiguity and an excessive resource investment strategy to protect the manager’s resources. When facing an ambiguous work context, work addiction could emerge as first-level healthcare managers work excessively and try to reduce the experience of guilt while not working (Spence and Robbins, 1992; Clark et al., 2016), knowing their team is lacking the information and support needed to function correctly (Kurtessis et al., 2017). Furthermore, role ambiguity is a structural problem emerging from a lack of clarity from top management. Thus, for managers, employees become a resource which requires defending from such ambiguity, and not a resource to support them. As such, we propose that under high role ambiguity and LMX, managers will aim to protect the valued relationship with their team, exacerbating the process leading to burnout. In fact, a manager will not be inclined to invest resources to support members of his team faced with ambiguity at work if he perceives his relationship with them as being unenriching.

The present article sheds light upon the process whereby role ambiguity incentives risky and excessive resources investment in the form of work addiction among first-level managers, leading to burnout. In this context, we contribute in three ways. First, we expand the nomological network of role ambiguity by testing work addiction as a mediator to explain variations in burnout among managers, and so, from a resource perspective. Previous research relating to role ambiguity and burnout has mainly focused on their direct relationship (Yürür and Sarikaya, 2012), thus not offering opportunities to reflect and theorize on the underlying mechanisms at play, such as work addiction (Clark et al., 2016). Furthermore, building upon the COR theory (Hobfoll, 1989, 2001), we investigate the existence of deleterious resource investment and protection strategies, an understudied but important occupational phenomenon. Second, we test under which conditions high LMX can have negative effects for managers. This potential “dark side” manifests as they overinvest themselves to protect their quality relation with their subordinates. As highlighted by Premru et al. (2022) in a recent literature review regarding LMX, this concept and its negative aspects have mainly been scrutinized from a subordinate standpoint. However, as a dyadic phenomenon, it should be studied from both perspectives to allow a more nuanced and complete overview. Indeed, by considering the ongoing resource investment needed by managers to reach high-quality relationships, we highlight the importance of their standpoint (Uhl-Bien et al., 2022). This new perspective on LMX both values the perceptions of the leaders and provides a boundary condition under which it does not provide positive outcomes. Third, we investigate a specific population which received little academic attention: first-level healthcare managers. They represent a population at risk of work addiction (Atroszko and Atroszko, 2020) and burnout (Delaye and Boudrandi, 2010) considering their complex role and the intense job demands they must face daily. We hope to bring more empirical attention to this population which plays a key role in healthcare management and call upon other researchers to do the same.

## Theoretical development and hypotheses

Role ambiguity, defined as the lack of clarity and predictability regarding one’s responsibilities towards others and the organization (Katz and Kahn, 1978), is a well-known stressor in the healthcare sector (e.g., Tunc and Kutanis, 2009; Yürür and Sarikaya, 2012). It is known to be positively related to depression (Schmidt et al., 2012), which in turn can have adverse effects on patients (Firth-Cozens, 2001). On the contrary, role clarity is known to have beneficial effects regarding well-being in various healthcare settings (Brunetto et al., 2011). Of note, the present article focuses exclusively on role ambiguity, eschewing other role stressors as antecedents. While they are also relevant in healthcare settings (Kilroy et al., 2016), we aim to study the specific impact of the lack of clarity regarding expectations among first-level managers. As professionals integrating managerial roles, their objectives and available resources at their disposal might be ambiguous and thus act as a salient stressor.

According to the COR theory, individuals need to protect and invest their resources to thrive (Hobfoll, 1989). More precisely, resources are “personal, material, energy and condition resources” which support goal attainment (Hobfoll et al., 2018, p. 113). Within a resource perspective, role ambiguity acts as a negative resource passageway, an ensemble of “ecological conditions that either foster and nurture or limit and block resource creation and sustenance” (Hobfoll et al., 2018, p. 106), as worthwhile investments are difficult to identify (Hobfoll, 2011). Indeed, in absence of clear goals and responsibilities, first-level managers have more difficulty allocating their resources (e.g., skills, knowledge, experience) and determining the proper priorities (Harvey and Kudesia, 2023). Ambiguous goals and lack of information as to which resource investment strategy is worth pursuing in demanding work contexts put managers at risk of having poor return on investment, threatening their resource pool (Shin et al., 2020). Such resource loss spirals can gain in magnitude for every investment with poor returns as managers try to recoup resources in an environment that does not provide them with proper information (Hobfoll, 2011).

According to the primacy of loss principle of the COR theory (Hobfoll et al., 2018), the process of resource loss is stressful for individuals. Prolonged experience of resource loss, and ensuing stress, is related to exhaustion in the form of burnout (Maslach et al., 1997). We propose that role ambiguity threatens managers’ resources, exhausting them, and ultimately results in burnout (Hobfoll et al., 2018).

*H1*: Role ambiguity is positively related to burnout.

When managers experience role ambiguity, excessive investment in one’s work can be used as a way to cope with unclear objectives. Under such conditions, work addiction can emerge from working more than expected and investing unreasonable levels of resources at work (Andreassen et al., 2012; Griffiths et al., 2018). Work addiction is often operationalized as “as being overly concerned about work, being driven by an uncontrollable work motivation, and spending so much energy and effort on work that it impairs private relationships, spare-time activities and/or health” (Andreassen et al., 2012, p. 265). In this context, as role ambiguity hinders efficient resource investment (Hobfoll et al., 2018), work addiction can become a maladaptive response from managers who aim to both reach their own uncertain goals and invest in their team by providing them with information and guidance.

Work addiction also manifests itself through feelings of guilt and anxiety while not working (Andreassen et al., 2012; Andreassen, 2014). Such negative feelings are most significant for managers, who are a population at risk of experiencing guilt (Schaumberg and Flynn, 2012), notably related to their perceived duty to shield their team from the adverse effects of an ambiguous work context (Clark et al., 2016; Griffiths et al., 2018). By attempting to diminish said guilt, managers may feel compelled to return to work, even if they lack the resources to do so. Furthermore, this uncontrollable need to work is especially meaningful in healthcare settings (Maisonneuve et al., 2024), as first-level managers dedicate themselves to supporting both their team and patients (Xie et al., 2023).

The excessive amount of resource invested at work to overcome an ambiguous work environment and the experience of anxiety in non-work contexts exposes managers to resource loss spirals (Hobfoll et al., 2018), exhausting them, and thus increasing the risk of burnout (Maslach and Jackson, 1981). In this context, work addiction becomes a significant threat to both the physical and mental well-being of first-level healthcare managers as they spend more time working and cutting leisure time to complete their tasks (Griffiths et al., 2018), leading to burnout (Engelbrecht et al., 2020; Sun et al., 2022). Previous studies have identified the organization of work as an antecedent to work addiction (Huyghebaert et al., 2018); while others identified work addiction as an antecedent to burnout among individuals working in healthcare (Schaufeli et al., 2009). Moreover, experiencing even low levels of work addiction was associated with increased emotional exhaustion (Gillet et al., 2017), the core dimension of burnout (Seidler et al., 2014). In the light of the cited findings and the principles of the COR theory, we propose that work addiction is an underlying mechanism whereas compensating for an ambiguous role can lead to burnout (Clark et al., 2016) by threatening individuals’ resource pools.

*H2*: Work addiction mediates the positive relationship between role ambiguity and burnout.

Following the formulated hypotheses, we investigated a boundary condition under which the relationship of the managers with their team may alter the proposed process. LMX, defined as “the quality of exchange between a leader and their subordinate” (Premru et al., 2022, p. 2), is often used to study the perception of such relationships. High LMX can have beneficial effects for subordinates (Dulebohn et al., 2012; Serban et al., 2021), such as reduced role overload (Tordera et al., 2008), increased performance (Martin et al., 2016), and stronger affective commitment (Montani et al., 2017). Additionally, LMX is most valuable when ambiguity is high as it provides them with important resources like information and support (Dunegan et al., 2002; Zhang et al., 2020). As for leaders, when they do develop good relationships with their team members, research indicates this can protect them from emotional exhaustion and depersonalization (Lai et al., 2018). However, high LMX is not intrinsically positive for the subordinates (Salehzadeh, 2020), their organization (Ballinger et al., 2010), and we add, first-level managers. Few studies have examined the opportunity cost for managers in respect to providing such resources to their team members. Working as a first-level manager requires significant resource investments (i.e., time and energy) to develop constructive LMX (Tremblay et al., 2021). Once built, maintaining quality exchanges requires ongoing investment (Nahrgang and Seo, 2015). This is in line with the core tenet of the COR theory which suggests that individuals protect what they value (Hobfoll, 1989; Halbesleben et al., 2014).

We know little about the effects of LMX in an ambiguous context on first-level managers’ work behaviors. In this specific context, role ambiguity arises as an issue stemming from inadequate clarity regarding objectives and expectations presented by the supervisors of first-level managers (Katz and Kahn, 1978). As the organization fails to provide explicit information, first-level managers must invest their time and effort in acquiring it from relevant sources (i.e., upper management). Thus, team members are not a resource for first-level managers when lacking clear objectives. They are, however, a resource regarding other goal attainment (Uhl-Bien et al., 2022), such as providing care for patients in healthcare settings. This context creates a double bind for first-level managers experiencing role ambiguity and perceiving high quality exchanges with their team members as they must invest resources to protect themselves and their team from ambiguity. Acquiring information and delivering it to their team to shelter its members from role ambiguity is no small feat (and resource investment) for managers. Such role stress nurtures overinvestment into work and limits managers’ ability to spend time recovering the expended resources (Sonnentag, 2018).

**Figure 1:**
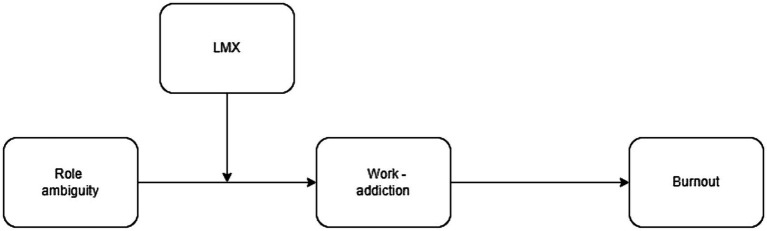
Theoretical model.

Following these observations, we posit that the interaction of high ambiguity and high LMX could create a “dark side” of resource protection, where first-level managers are motivated to pathologically devote themselves to work and develop work addiction as a maladaptive resource investment strategy. This approach, in turn, fosters burnout as resources are spent faster than they are recouped (Halbesleben et al., 2014). Thus, LMX will alter the relationship between role ambiguity and both work addiction and burnout. With the present theoretical model, we propose a new perspective regarding the necessary resource investment from managers who perceive quality exchanges with their team members and how this could potentially affect them in a negative way. Figure 1 presents the tested theoretical model.

*H3a*: LMX moderates the relationship between role ambiguity and work addiction such that the higher the LMX, stronger is the relationship.

*H3b*: LMX moderates the indirect relationship between role ambiguity and burnout such that the higher the LMX, stronger is the relationship.

## Methods

### Research design

The cross-sectional data for this study was collected using an open questionnaire hosted by Qualtrics. We received support from two associations representing the interests of managers working across multiple organizations in healthcare in the public sector in eastern Canada to distribute the questionnaire. Before responding, all participants were required to read and sign a consent form, granting them the opportunity to modify their responses and withdraw their participation at any given moment. No incentives were provided to participants.

### Measures

First, role ambiguity was measured using the scale developed by Rizzo et al. (1970). It is composed of 6 items on a 7-point Likert scale (1 = Totally disagree to 7 = Totally agree) and all items are reverse coded (Schuler et al., 1977). Sample item is “I have clear, planned goals and objectives for my job.” Second, work addiction was measured using the Bergen Work Addiction Scale (BWAS) developed by Andreassen et al. (2012). It is composed of 7 items rated on a temporal anchoring (1 = Never to 5 = Always). Sample item is “I prioritise work over hobbies, leisure activities, and exercise.” Third, burnout was measured using the short scale developed by West et al. (2012) rated on a 7-point Likert scale (1 = Totally disagree to 7 = Totally agree). It is composed of two items, one for emotional exhaustion and one for depersonalization. Items are “I feel burned out from my work” and “I have become more callous toward people since I took this job.” Fourth, LMX was measured with the LMX7 scale developed by Graen and Uhl-Bien (1995). It is composed of 7 items rated on a 7-point Likert scale (1 = Totally disagree to 7 = Totally agree). Sample item is “I understand my team members’ problems and needs.”

We introduced five control variables: age, gender, organizational tenure (measured in years), average worked hours per week, and role overload. Age was selected as previous results regarding age and burnout are not fully conclusive (Marchand et al., 2018). Controlling for age thus appears relevant to better contextualize the relationships between our variables of interest. Gender is often controlled for in research using healthcare sample as there is a systematic overrepresentation of women. This is especially true when studying burnout as women score higher than men (Jourdain and Chênevert, 2010). Gender was measured as a sociocultural factor, with the following options: cis woman, cis man, genderqueer, trans woman, trans man, prefers not to answer. An “additional category, please specify” was added to insure maximum flexibility in responses. Tenure was selected as it has long been known to influence work attitudes (see Salancik and Pfeffer, 1978). Hours per week were added to better isolate the effect of work addiction (Griffiths et al., 2018; Griffiths, 2024). Role overload was added to control for its impact on burnout and was measured using Brown et al.’s (2005) 4-item measure rated on a 7-point Likert scale (1 = Totally disagree to 7 = Totally agree).

## Results

Data collection spanned from September 2022 to March 2023 with information regarding the project and an invitation to complete the questionnaire being shared with members of two interprofessional associations of managers in the healthcare sector. After data curation, only 165 had fully completed our survey, which represents an estimated 12% response rate. Table 1 presents the demographic statistics of the participants who completed the questionnaire.

**Table 1:** Demographics.

Variables (*N* = 165)	Frequency	Percentage
*Age*		
Less than 30	3	1.8
30 to 39	29	17.5
40 to 49	66	40.0
50 to 59	58	35.2
60 and up	9	5.5
*Gender*		
Woman	123	74.5
Man	33	20.0
Prefers not to answer	9	5.5
*Tenure*		
1 to 9	55	33.3
10 to 19	52	31.5
20 to 29	37	22.5
30 and up	21	12.7
*Hours per week*		
Less than 40	10	6.1
40 to 49	84	50.8
50 to 59	58	35.2
60 and up	13	7.9

Due to the cross-sectional and self-reported nature of our data, we tested if common method bias could prove to be problematic (Podsakoff et al., 2003). First, we used the Harman’s single factor test, which consists of conducting an exploratory factor analysis with maximum likelihood in an unrotated structure and fix the number of factors to one. The obtained factor explained only 20.5% of the variance, below the 50% threshold (Malhotra et al., 2006). Second, using a linear regression model, all observed variance inflation factors were below 1.5, which is under the strict threshold of 2.5 (Johnston et al., 2018). Overall, both tests indicated that common method bias does not appear to be of major concern, and thus we proceeded with the main statistical analyses. Table 2 presents the means, standard deviations (SD), correlations, and alphas on the diagonal.

**Table 2:** Means, SD, correlations and α.

Variables	Mean	SD	1	2	3	4	5	6	7	8	
1. Role ambiguity	2.81	0.98	(0.85)								
2. Work addiction	3.02	0.78	0.29**	(0.84)							
3. Burnout	4.21	1.54	0.29**	0.61**	(0.71)						
4. LMX	5.91	0.71	−0.17*	0.09	0.01	(0.84)					
5. Age	46.88	8.09	−0.23**	−0.19*	−0.16*	0.02	–				
6. Gender	–	–	0.17*	−0.07	0.20*	−0.19*	0.09	–			
7. Tenure	15.45	10.24	−0.19*	−0.09	−0.15	−0.01	0.43**	0.03	–		
8. Hours per week	47.02	7.01	0.06	0.42**	0.31**	0.05	−0.04	0.01	0.09	–	
9. Role overload	5.44	1.26	0.21**	0.54**	0.58**	0.01	−0.10	0.15	−0.04	0.28**	(0.90)

Scale internal consistency (α) reported on the diagonal.

Two-tailed, *p < 0.05; **p < 0.01. Gender coded 0 = woman, 1 = man.

*N* = 165 (gender has 9 missing data).

Before testing the hypotheses, we conducted a confirmatory factor analysis (CFA) using AMOS 28. The model fit indices indicated a satisfactory factorial structure (Bentler and Bonett, 1980): χ^2^ = 353.33 (df = 202, *p* < 0.001), comparative fit index (CFI) = 0.91, adjusted goodness of fit (AGFI) = 0.81, root mean square error of approximation (RMSEA) = 0.07, standardized root mean square residual (SRMR) = 0.07. We then tested the 4-factor model against more parsimonious model. 3-factor model I collapsed burnout with work addiction, 3-factor model II collapsed work addiction with role ambiguity, and then the 1-factor model loaded all items on a single latent factor. Considering the similar fit indices provided by 3-factor model I to the 4-factor model, we conducted a chi-square difference test (Δχ^2^ = 14.08, df = 3, *p* < 0.01), and results indicated a significant difference. Model fit indices comparisons are presented in Table 3. As results demonstrated significant differences in model fit indices, we maintained our proposed 4-factor model and proceeded to the regression analyses.

**Table 3:** Model fit indices comparisons.

Model	CFI	AGFI	RMSEA	SRMR
4-factor model	0.91	0.81	0.07	0.07
3-factor model I	0.90	0.80	0.07	0.08
3-factor model II	0.70	0.55	0.12	0.14
1-factor model	0.44	0.40	0.16	0.19

Using the PROCESS macro (Hayes, 2017) in SPSS 28, we tested the moderated mediation model (Model 7) using a 5,000 bootstrap-sample with a 95% confidence interval. All variables were standardized before analysis to provide comparable effect sizes. However, this method does not allow missing data to proceed with the regressions. Considering that 9 respondents did not answer regarding their gender, they were treated as missing data. To convey all results in a transparent and coherent manner, results for Model 1 represents the standardized regression weights without the control variables (*N* = 165) and Model 2 with the control variables (*N* = 156). This approach allows to better contextualize the data.

Starting with Model 2 and the control variables, average hours per week (*β* = 0.31, *p* < 0.001) and role overload (*β* = 0.41, *p* < 0.001) were significantly related to work addiction. These results are in accordance with previous empirical studies (Andreassen, 2014; Clark et al., 2016). Furthermore, role overload was also associated with burnout (*β* = 0.29, *p* < 0.001), which is also coherent with the literature on the subject. Gender was associated both with work addiction (*β* = −0.15, *p* = 0.020) and burnout (*β* = 0.18, *p* = 0.005). While the first result is not out of the ordinary, despite mixed findings regarding gender and work addiction (Clark et al., 2016), the fact that the coefficient was positive for burnout is surprising. Usually, samples present women as more prone to burnout (Brady et al., 2021). Considering that our sample is composed exclusively of managers, perhaps this could explain the result. Men in a managerial position could be more prone to express the frequency of their burnout symptoms, thus explaining the observed result.

For the hypotheses, it is important to note that Model 1 and Model 2 yielded the same conclusions, with only the regression weights and R2 being modified by the inclusion of control variables. This demonstrates the rigorousness of the theoretical model. The full results of the two models are presented in Table 4. As for the hypotheses tested below, we present results from Model 2.

**Table 4:** Full model results.

	Work addiction		Burnout	
Variables	*β* Model 1	*β* Model 2	*β* Model 1	*β* Model 2
*Control variables*				
Age	–	−0.09	–	−0.04
Gender	–	−0.15*	–	0.18**
Tenure	–	−0.04	–	−0.09
Work hours	–	0.31**	–	−0.07
Role overload	–	0.41**	–	0.29**
*Direct effects*				
Role ambiguity	0.30**	0.18**	0.12	0.05
Work addiction	–	–	0.58**	0.41**
LMX	0.21**	0.12	–	–
RA × LMX	0.28**	0.18*	–	–
*Indirect effects*				
Role ambiguity	–	–	0.18*	0.08*
RA × LMX	–	–	0.16*	0.07*
R2	0.16	0.47	0.39	0.50

Model 1 *N* = 165; Model 2 *N* = 156. *p < 0.05; **p < 0.01 (two-tailed).

RA, role ambiguity.

H1 posited that role ambiguity was positively related to burnout. The direct effect was not significant (*β* = 0.05, *p* = 0.455), thus H1 was not supported. H2 proposed that work addiction mediated the indirect effect of role ambiguity on burnout. The indirect effect was significant and in the predicted direction (*β* = 0.08, 95% CI [0.019, 0.141]), providing support to H2. In conjunction with the result from H1, we thus observed an indirect-only mediation (Zhao et al., 2010).

**Figure 2:**
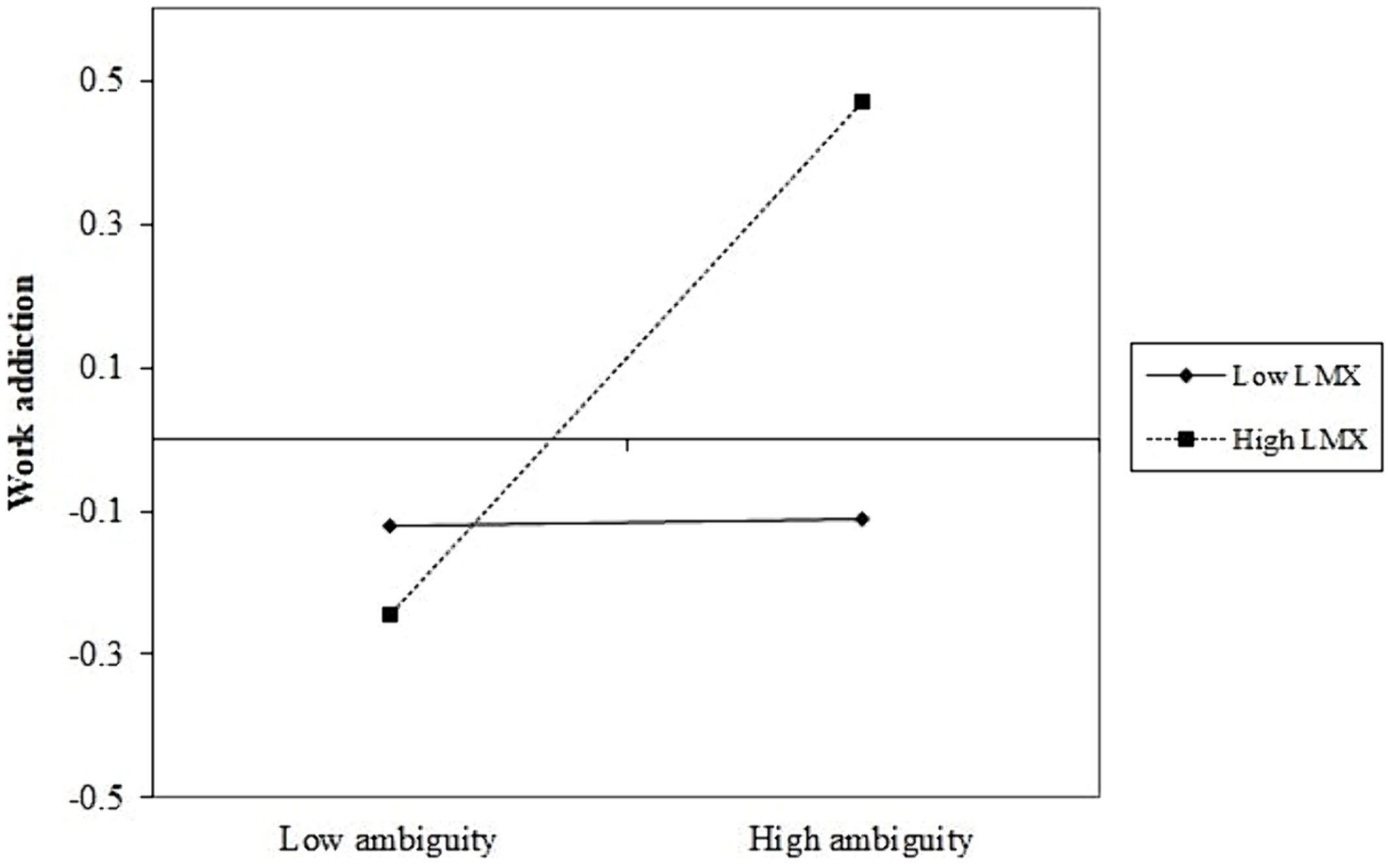
Two-way interaction.

H3a proposed that LMX moderated the direct relationship between role ambiguity and work addiction such that the relationship was stronger when LMX was high. The direct effect of the interaction term was significant and in the predicted direction (*β* = 0.18, *p* = 0.023). To probe the interaction further, we tested it at low (−1 SD) and high (+1 SD) levels of LMX. Results indicated that the relationship was stronger when LMX was high (*β* = 0.36, 95% CI [0.16, 0.56], *p* < 0.001), and became non-significant when LMX was low (*β* = 0.01, 95% CI [−0.19, 0.21], *p* = 0.910); supporting H3a. Figure 2 presents the interaction (Dawson, 2014).

H3b proposed that LMX moderated the indirect relationship between role ambiguity and burnout mediated by work addiction such that the relationship was stronger when LMX was high. First, the index of moderated mediation was significant (0.072, 95% CI [0.004, 0.147]). We thus probed the interaction further. Second, the indirect effect was stronger when LMX was high (*β* = 0.147, 95% CI [0.054, 0.252]), and became non-significant when LMX was low (*β* = 0.005, 95% CI [−0.084, 0.092]). Third, the pairwise comparison between the two conditional indirect effects was significant (0.143, 95% CI [0.009, 0.289]). These results lend support to H3b. Table 4 summarizes the results.

## Discussion

To the best of our knowledge, this study is among the first to explore the potential negative impacts of LMX from the leader’s perspective. Previous studies looked at the relationship between LMX and work addiction (see Afota et al., 2021), but as an antecedent among subordinates. We focused our attention on a population, first-level healthcare managers, whose intrinsic role potentially puts their well-being at risk. First-level managers often face significant demands without the necessary resources, fostering work addiction (Atroszko and Atroszko, 2020) and burnout (Delaye and Boudrandi, 2010). Despite these risks, managers remain widely understudied compared to their subordinates, especially in healthcare settings. The healthcare sector in Canada is composed at 75% of women (Khanam et al., 2022), which informs us that the observed results disproportionally affect women. Access to such a population for research remains a challenge for organizational behavior, human resources management, and leadership scholars alike.

Regarding the results, we observed that work addiction fully mediated the relationship between role ambiguity and burnout. This is interesting as both role ambiguity and work addiction have been identified as direct antecedents to burnout (Yürür and Sarikaya, 2012; Clark et al., 2016, respectively), but the meditation process was never tested. This finding informs us that role ambiguity, theorized as a negative resource passageway (Hobfoll, 2011; Halbesleben et al., 2014), hinders the ability of managers to invest their resources in a constructive and efficient manner as they pursue disadvantageous investments. Such a context entices managers to invest themselves excessively in their work in order to accomplish a plurality of potentially relevant goals. Accordingly, they devote pathological levels of time and effort to their work, to the point of cutting leisure time and damaging their emotional and physical health (Andreassen et al., 2012). These excessive behaviors drain managers of their resources, as low or absent returns on investments initiate resource loss spirals, in turn leading to burnout. Understanding this process is paramount as the issue of burnout is already highly prevalent among managers (Wu et al., 2019), and especially among individuals in healthcare settings (Khamissa et al., 2022).

Additionally, high LMX while facing role ambiguity exacerbates work addiction and burnout among managers. Indeed, high quality LMX, from the perspective of the leader, represents a valuable resource which deserves protection. As such, first-level healthcare managers overexert themselves to compensate the negative repercussions of ambiguity. This fosters an unhealthy relationship with work in the form of work addiction as managers work too much without proper mechanisms to replenish their resources (Sonnentag, 2018). In turn, this addiction to work is related to higher levels of burnout as emotional exhaustion and depersonalization settle in. Despite being a supportive resource for managers in other contexts, high LMX acts as a catalyst of negative outcomes when navigating an ambiguous work environment. On the contrary, when LMX is low, role ambiguity has no relationship with work addiction, as first-level managers do not feel the compulsion to protect their team from such a context. They do not perceive their team as a resource worth defending. Furthermore, low LMX nullifies the indirect relationship between role ambiguity and burnout as first-level managers do not overexert themselves at work. Perceiving the exchanges with their team members as unhelpful, they do not invest themselves neither into developing or protecting it. These findings provide useful implications regarding both theory and practice.

### Theoretical implications

First, we bring a more nuanced perspective to LMX by adopting a COR perspective. Our results highlight that perceiving high-quality relationships with team members is not inherently positive for managers. This offers both a nuanced and new perspective to the LMX literature, which often centers around the perceptions of and benefices for the subordinates. Theorizing LMX as a resource which requires initial investment to emerge as such within the COR theory opens new research avenues regarding its evolution within a team and how it influences the resource investment strategies of its members. Indeed, few previous studies considered the required resource investment from leaders to provide, for example, information to their members to decrease their role ambiguity (Jian, 2014). Furthermore, our findings indicated that low LMX protected managers from work addiction under ambiguous work conditions. This observation is in no shape or form a justification to value low LMX, but on the contrary, a plea towards more research regarding LMX from the standpoint of leaders. Overall, we contribute to the LMX literature by exposing a situation from the point of view of the leader where the “dark side” of a positive relationship with its team emerges. Managers being driven to work pathologically to the detriment of their own well-being, to support their team, appears to be an important limit to high LMX and deserves further scientific inquiry.

Second, we contribute to the COR theory by demonstrating that a lack of clarity regarding goals pushes managers towards risky and excessive resource investment strategies which in turn is related to higher levels of exhaustion. As resources investment becomes less efficient due to the lack of information, managers must compensate by overinvesting themselves, ultimately experiencing resource loss spirals. Relatedly, our results indicated that work addiction acted as the underlying mechanism regarding the relationship between role ambiguity and burnout. While direct relationships between the constructs were previously studied (Shin et al., 2020; Sun et al., 2022), we contribute by adding nuance to role ambiguity’s nomological network. Role ambiguity does not in itself lead managers to burnout, but it does so indirectly, by having a deleterious effect on resource investment at work, enticing first-level healthcare managers to work excessively. Spending pathological time and effort doing and thinking about work exhausts resources (Clark et al., 2016), which is related to higher burnout. This process emphasizes the value of role clarity not only to protect the individual resources of first-level managers, but also as a protective factor regarding their relationship with work itself.

Third, as highlighted by our results, the number of worked hours had a non-significant direct relationship with burnout, while work addiction did. The implication of this finding is that work addiction is not merely an amount of time spent working (Griffiths, 2005), but a pathological relationship with work which yields negative outcomes. Work addiction is indeed related to individuals working more hours, but most importantly, to investing more effort, on sometimes dubious objectives, while also draining resources outside of work in the form of negative feelings. The expenditure of excessive resources at work combined with a lack of proper recovery during non-work is what makes work addiction intrinsically harmful in nature (Andreassen et al., 2019) beyond worked hours.

### Practical implications

While previous studies have focused on the role managers play regarding the occupational health of their team members (Thomas and Lankau, 2009), less is known regarding the multiple challenges that managers must address to meet their managerial responsibilities. Indeed, first-level managers can often face intense and poorly operationalized demands originating either from their superiors or their team. Accordingly, the context in which first-level managers operate is crucial as it can nurture or hinder suitable resource investments (Halbesleben et al., 2014), and so towards both themselves and their subordinates. In this study, we highlighted the importance of reducing an important contextual factor, namely role ambiguity, as it was related to work addiction, and in turn burnout, among first-level healthcare managers. Therefore, providing role clarity among this population emerges as a priority for healthcare organizations as it should act as a protective factor against deleterious resource overinvestment (Brunetto et al., 2011). This implies various complementary measures originating from multiple stakeholders in the organization, such as higher management and HR practitioners.

First, considering that many first-level managers in healthcare are formally trained as caregivers, not in management. Without proper training and support from their organization, they are particularly at risk of experiencing ambiguity. Due to potentially competing interests and objectives present in healthcare settings (Yoshie et al., 2008), providing formal career development strategies including onboarding and training for the acquisition of a managerial role should emerge as a priority. Accordingly, healthcare organizations, with the support of their human resources departments, should not only emphasize the various expectations associated with the different roles that managers have to assume daily, but also provide support and coaching in the roll-out of managerial training to promote role clarity among first-level managers. The aim of these initiatives is not to stifle autonomy among managers, but to provide explicit and contextualized goals, methods, and information regarding formal authority granted by the role of first-level manager.

Second, healthcare organizations should improve the quality and quantity of their communication practices to reduce the role ambiguity of first-level managers. This applies for both top-down information practices—received by first-level managers and transmitted to subordinates—and for bottom-up information diffusion—received by first-level managers and transmitted to higher management and HR teams. Therefore, appropriate top-down information flow could help first-level managers to develop a better understanding of the context and the strategic orientations supported by both the organization and the health ministry. In turn, this would allow managers to draw a clear line regarding the aligned operational actions that should be implemented. In addition, the existence of bottom-up communication channels could favor a better grasp of the operational issues for first-level managers that would act as a fertile ground to bring up necessary adjustments to higher organizational level. In summary, this would reduce uncertainty and opacity regarding their expected behaviors, goals to attain, and the decision-making processes, thus promoting appropriate resource investments for first-level managers that will protect them from negative consequences.

### Limits and future research

Despite the significant contributions of our study, it is important to acknowledge its limitations, which offer directions for future research. One primary limitation is the context-specific nature of our findings. Given the unique characteristics of Canada’s healthcare system (e.g., public, regulation; resource availability, regulation surrounding the worked hours), our results may not be directly generalizable to other work settings or even other healthcare sectors. This may be because these characteristics can influence the operational framework and regulatory environments that influence managers’ behaviors, engagement, and service delivery. Additionally, our sample, although representative of the Canadian healthcare sector, exhibits an overrepresentation of women. This gender distribution, while reflective of our study’s context, may not represent the workforce composition in other industries, potentially affecting the generalization of our findings. Another constraint is the size of our sample. While our study taps into an understudied population, offering fresh perspectives and contributing to the literature, the relatively small sample size may limit the statistical power of our findings. Furthermore, the cross-sectional nature of our data collection restricts our ability to infer causality or track changes over time. Additionally, the cross-sectional and self-reported nature of our design may lead to the potential for common method bias, despite our analysis suggesting otherwise. This bias might lead to inflated associations between variables due to the shared method of data collection rather than genuine relationships.

Following these limits, futures studies could replicate or build upon the findings. On one hand, researchers aiming to replicate the proposed model should focus on national or international sampling. What is considered a good LMX varies across cultures, and so testing the model outside of North America could provide valuable insights on the perception of managers regarding their obligation to their team. Furthermore, healthcare systems vary widely across the globe, reinforcing the importance of testing the proposed relationships in other settings to deepen our understanding of this process specifically for healthcare managers. Additionally, replicating the model with longitudinal data could provide valuable insight regarding the observed relationships and test alternative models using random intercept cross lagged panel modeling for example.

To build upon the findings, first, we propose a call for research regarding other role stressors and their impact on work addiction as a maladaptive coping mechanism among first-level managers. Overall, more research regarding this population is needed to properly help organizations support their first line of management. Second, more research should investigate the “dark side” of LMX for managers and how developing this resource, while very beneficial for team members (Premru et al., 2022), can be a costly process for them. Organizations should strive to have healthy managers and reduce their potential inclination to sacrifice themselves to protect their valued team members from an adversarial work context. In addition, future research could focus on the theorization of low perceived LMX by employees. A potential theorization would be that managers experiencing resources loss or exhaustion refrain from investing into their relationships with their team members to preserve resources. As such, employees would perceive lower LMX as a result of a withdrawal behavior when resources are scarce (e.g., Groulx et al., 2024). However, we cannot address this question with our current research design. We underscore the necessity for further research, particularly studies employing longitudinal designs, to elucidate the causal relationships between these variables. Longitudinal studies would allow for the examination of changes over time of LMX providing insights into how the relationship between exhaustion and LMX develops and evolves. Third, exploring the observed relationships in other sectors could provide valuable insights regarding the generalizability of the findings. For example, little is known regarding work addiction or burnout among managers in the manufacturing sector, despite knowledge that this sector has important psychosocial risk factors (Madnawat and Mehta, 2012). Future inquiry in this sector with both quantitative and qualitative methods could shed light on the specific conditions of manufacturing organizations and their impact on managers.

## Conclusion

In this study, we observed that high LMX can act as a catalyst for work addiction among first-level managers experiencing role ambiguity. By attempting to shelter their team from unclear expectations and goals, first-level healthcare managers invest inordinate amounts of resources, like time and effort. Work addiction fully mediated the positive relationship between role ambiguity and burnout in this population. Additionally, the mediation was exacerbated when LMX was high and disappeared when low. These results are not an invitation to value low LMX among managers, but an indication for healthcare organizations to provide clear directives to their managers to foster well-being at work. Reducing role ambiguity should provide a positive work context, in the form of constructive resource passageway, which frees first-level managers’ resources to care for themselves, their team, and in turn, patients.

## Data availability statement

The raw data supporting the conclusion of this article will be made available by the authors, without undue reservation.

## Ethics statement

The studies involving humans were approved by Comité d’éthique en recherche HEC Montréal (Project number: 2022-4467). The studies were conducted in accordance with the local legislation and institutional requirements. The participants provided their written informed consent to participate in this study.

## Author contributions

FM: Writing – review & editing, Writing – original draft, Visualization, Project administration, Methodology, Investigation, Formal analysis, Data curation, Conceptualization. PG: Writing – review & editing, Writing – original draft, Methodology, Formal analysis, Conceptualization. AG: Writing – review & editing, Writing – original draft, Conceptualization. DC: Validation, Writing – review & editing, Supervision, Investigation, Funding acquisition. MC: Project administration, Writing – review & editing, Supervision, Investigation, Funding acquisition.
